# Comprehensive analysis and establishment of a prediction model of alternative splicing events reveal the prognostic predictor and immune microenvironment signatures in triple negative breast cancer

**DOI:** 10.1186/s12967-020-02454-1

**Published:** 2020-07-28

**Authors:** Shanshan Yu, Chuan Hu, Lixiao Liu, Luya Cai, Xuedan Du, Qiongjie Yu, Fan Lin, Jinduo Zhao, Ye Zhao, Cheng Zhang, Xuan Liu, Wenfeng Li

**Affiliations:** 1 Department of Chemoradiation Oncology, The First Affiliated Hospital of Wenzhou Medical University, 2 Fuxue Road, Wenzhou, Zhejiang 325000 People’s Republic of China; 2 Department of Orthopaedic Surgery, the Affiliated Hospital of Qingdao University, Qingdao, 266071 China; 3 Department of Obstetrics and Gynecology, The First Affiliated Hospital of Wenzhou Medical University, Wenzhou, Zhejiang People’s Republic of China; 4 Department of Dermatology, The First Affiliated Hospital of Wenzhou Medical University, Wenzhou, Zhejiang People’s Republic of China

**Keywords:** Alternative splicing, TNBC, Nomogram, Prognosis, Immune signatures

## Abstract

**Background:**

Triple-negative breast cancer (TNBC) is widely concerning because of high malignancy and poor prognosis. There is increasing evidence that alternative splicing (AS) plays an important role in the development of cancer and the formation of the tumour microenvironment. However, comprehensive analysis of AS signalling in TNBC is still lacking and urgently needed.

**Methods:**

Transcriptome and clinical data of 169 TNBC tissues and 15 normal tissues were obtained and integrated from the cancer genome atlas (TCGA), and an overview of AS events was downloaded from the SpliceSeq database. Then, differential comparative analysis was performed to obtain cancer-associated AS events (CAAS). Metascape was used to perform parent gene enrichment analysis based on CAAS. Unsupervised cluster analysis was performed to analyse the characteristics of immune infiltration in the microenvironment. A splicing network was established based on the correlation between CAAS events and splicing factors (SFs). We then constructed prediction models and assessed the accuracy of these models by receiver operating characteristic (ROC) curve and Kaplan–Meier survival analyses. Furthermore, a nomogram was adopted to predict the individualized survival rate of TNBC patients.

**Results:**

We identified 1194 cancer-associated AS events (CAAS) and evaluated the enrichment of 981 parent genes. The top 20 parent genes with significant differences were mostly related to cell adhesion, cell component connection and other pathways. Furthermore, immune-related pathways were also enriched. Unsupervised clustering analysis revealed the heterogeneity of the immune microenvironment in TNBC. The splicing network also suggested an obvious correlation between SFs expression and CAAS events in TNBC patients. Univariate and multivariate Cox regression analyses showed that the survival-related AS events were detected, including some significant participants in the carcinogenic process. A nomogram incorporating risk, AJCC and radiotherapy showed good calibration and moderate discrimination.

**Conclusion:**

Our study revealed AS events related to tumorigenesis and the immune microenvironment, elaborated the potential correlation between SFs and CAAS, established a prognostic model based on survival-related AS events, and created a nomogram to better predict the individual survival rate of TNBC patients, which improved our understanding of the relationship between AS events and TNBC.

## Introduction

Breast cancer is the most common cancer among women in the world, with the highest incidence, and is the second leading cause of cancer-related death, which has strong impacts on national economic and social development [1]. There are many kinds of breast cancers, among which triple negative breast cancer (TNBC) is a well-known subtype with high invasion, a low survival rate and a lack of effective treatment, accounting for 15–20% of all breast cancers and becoming an intractable problem in the breast cancer field [2]. The clinical manifestation of TNBC is an aggressive course, which is prone to local recurrence and distant metastasis. Once there is recurrence or metastasis, the median survival time is less than 1 year [3, 4]. The anti-programmed cell death (PD)-1 immunotherapy has taken on a favourable effect in TNBC according to the results of recent research, but the efficacy rate of these drugs is still low [5–7]. The poor prognosis of TNBC is closely related to its early high metastasis rate and recurrence rate [8–10], which suggests the urgent need to develop new biomarkers with high accuracy to predict the prognosis of TNBC patients.

In recent years, research on cancer genomics has gradually opened a new chapter benefiting from the tremendous development of bioinformatics and high-throughput technologies. A growing body of evidence shows that alternative splicing (AS) events play a pivotal role in the development of cancer and the formation of the tumour microenvironment [11, 12]. AS events have great influence in the process of mRNA precursor maturation, bringing about the splicing of different mRNA isomers and the translation of protein variants, which is one of the key regulatory mechanisms for the diversity of the transcriptome and proteome [13, 14]. In normal physiological processes, more than 95% of human genes undergo AS and encode splicing mutations [15]. AS not only exerts an important influence in normal physiological processes such as haematopoiesis and muscle [16, 17] but also plays a crucial role in carcinogenic pathological processes such as tumour proliferation, apoptosis, hypoxia, angiogenesis, immune escape and metastasis [18, 19]. Moreover, protein diversity leads to functional diversity, and quantitative change causes qualitative change. The production and accumulation of irregular AS events change the expression of some key splicing factors and promote the changes in the targeted AS parent genes, which together provide an advantage for the growth or survival of cancer cells [20]. Therefore, increasing amounts of attention have been paid to research on the effect of AS events on the cancer prognosis. The comprehensive analysis of AS events is expected to provide new potential biomarkers for the diagnosis and prognosis of cancer.

The prognostic value derived from AS events has been confirmed in a variety of cancer types, including liver cancer [21], head and neck tumours [22], kidney carcinoma [23], and pancreatic cancer [24]. However, to our knowledge, comprehensive analysis of AS signals in TNBC is lacking. Because of the severe prognosis of TNBC, it is urgent and necessary to carry out relevant research. In our study, we obtained and integrated transcriptome and clinical data of 169 TNBC tissues and 15 adjacent normal tissues from the cancer genome atlas (TCGA) and downloaded the data of AS events from the SpliceSeq database. Then, several bioinformatic and statistical methods were performed to analyse the function and prognostic value of AS events in TNBC patients, which filled in the blanks of TNBC in the aspect of AS events and laid a theoretical foundation for guiding clinical work and evaluating patient prognosis.

## Methods

### Data collection based on TCGA

We collected the transcriptome data and the clinical information of TNBC tissues and normal breast tissues from the TCGA data portal. We also downloaded data for AS events from the TCGA SpliceSeq database. There was a broad consensus that the goal of using Percent Spliced In (PSI) [25] ranging from 0 to 1 is to quantify events. We then set a strict set of screening conditions (sample percentage with a PSI value of 75 and an average PSI value of 0.05) to ensure the reliability of AS events included in subsequent analysis. Ultimately, 169 TNBC patients were included in our research.

### Screen for cancer-associated AS (CAAS) events between TNBC and normal tissues

To analyse the significantly differentially expressed AS events between tumour and normal samples, 169 TNBC and 15 normal tissues were used to perform differential analysis using the “limma” package. We took log FC (log twofold change) and false discovery rate (FDR) as the indexes to observe the expression differences, that is, associated adj.p values. We set the condition of | log2fc |≥ 1 and FDR/adjusted *P* < 0.05 to represent the upregulation and downregulation of relevant events, respectively.

### Functional enrichment analysis and exploration of the immune microenvironment

Based on the CAAS events, we further performed enrichment analysis of the corresponding parent genes. Gene Ontology (GO) and Kyoto Encyclopedia of Genes and Genomes (KEGG) pathway analyses were performed in metascape (www.metascape.org). The first 20 important pathways, if possible, were shown in the KEGG and GO analyses. Additionally, the TCGA TNBC cohort was classified by hierarchical consensus clustering. We employed the ConsensusClusterPlus package for the sake of clustering in an unbiased and unsupervised manner, and patients were classified into several clusters [26]. To obtain a robust classification, the optimal numbers of clusters were further validated according to the Elbow method and the Gap statistic. The differences in immune cells and the tumour immune microenvironment between the three clusters were compared by K-W test or the Wilcoxon rank-sum test.

### Building of potential SF–AS regulatory network

Studies have shown that splicing factor (SF) plays an indispensable role in regulating the development and progress of malignant tumors [27, 28].We downloaded SF data from the SpliceAid2 database (https://www.introni.it/splicing.html).Pearson correlation analysis was used to analyze the relationship between SFs expression and PSI values of CAAS events. Absolute value of correlation coefficient > 0.5, *P* < 0.001 is considered to be correlated.The correlation plot was generated by Cytoscape (version3.7.2).

### Construction of prognostic models and survival analysis

To further understand the prognostic value of AS events in TNBC patients, univariate Cox regression analysis was performed to determine survival-related DEAS events, including OS (overall survival)- and PFS (progression-free survival)-related DEAS events. Meanwhile, the UpSetR package in R was performed to draw two UpSet diagrams to show the interactions between seven types of survival-related CAAS events [29, 30]. Next, the least absolute shrinkage and selection operator (LASSO) regression was applied to identify the final elimination of potential predictors with non-zero coefficients [31], which can avoid model overfitting to obtain a better-fitting model. Furthermore, predictive models according to the results of LASSO Cox regression were constructed using multivariate Cox regression analysis. Based on PSI values and multivariate Cox analysis, we calculated the risk scores of each patient and obtained the corresponding coefficients, respectively. Risk score can be obtained by the following formula: $score ={\sum }_{i=0}^{n}PSI\times {\beta }_{i}$, where β is the regression coefficient.

A total of 169 TNBC patients were divided into high- and low-risk groups bounded by the median of risk score, and Kaplan–Meier survival analysis was performed to determine whether they had completely different prognoses. Furthermore, receiver operating characteristic (ROC) curves of 2, 3, and 4 years were generated to show the discrimination of predictive signatures.

### Development of an AS-clinic nomogram

A nomogram is an easy to use tool for clinical practice, especially in clinical oncology. Therefore, we utilized AS-based risk scores and clinical variables to develop 2 nomograms. First, we used univariate Cox analysis to screen corresponding variables related to survival, including OS and PFS. We utilized a forward stepwise variable selection with the Akaike information criterion (AIC) to filter the variables included in the final model nomogram. Finally, we created calibration curves to assess the predictive accuracy of the final nomogram and adopted the concordance index (C-index) as an index to quantify its discrimination capacity with the help of Hmisc package (version 4.1.1).

### Statistical analysis

All statistical analyses were performed in R software, version 3.6.1. All statistical tests with *p* < 0.05 (two-sided) were statistically significant.

## Results

### Identification of CAAS events

**Fig. 1:**
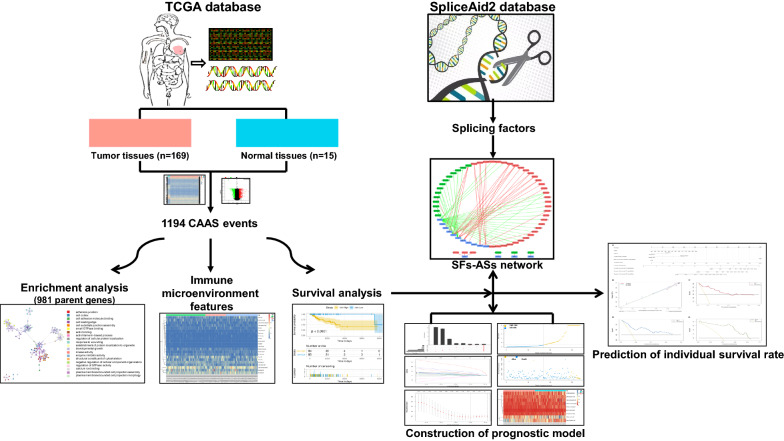
Flowchart of the systematic profiling of cancer-associated alternative splicing in TNBC in the study design.TCGA, The Cancer Genome Atlas; CAAS, cancer-associated alternative splicing; TNBC, triple negative breast cancer; AS, alternative splicing;SF, splicing factors

**Fig. 2:**
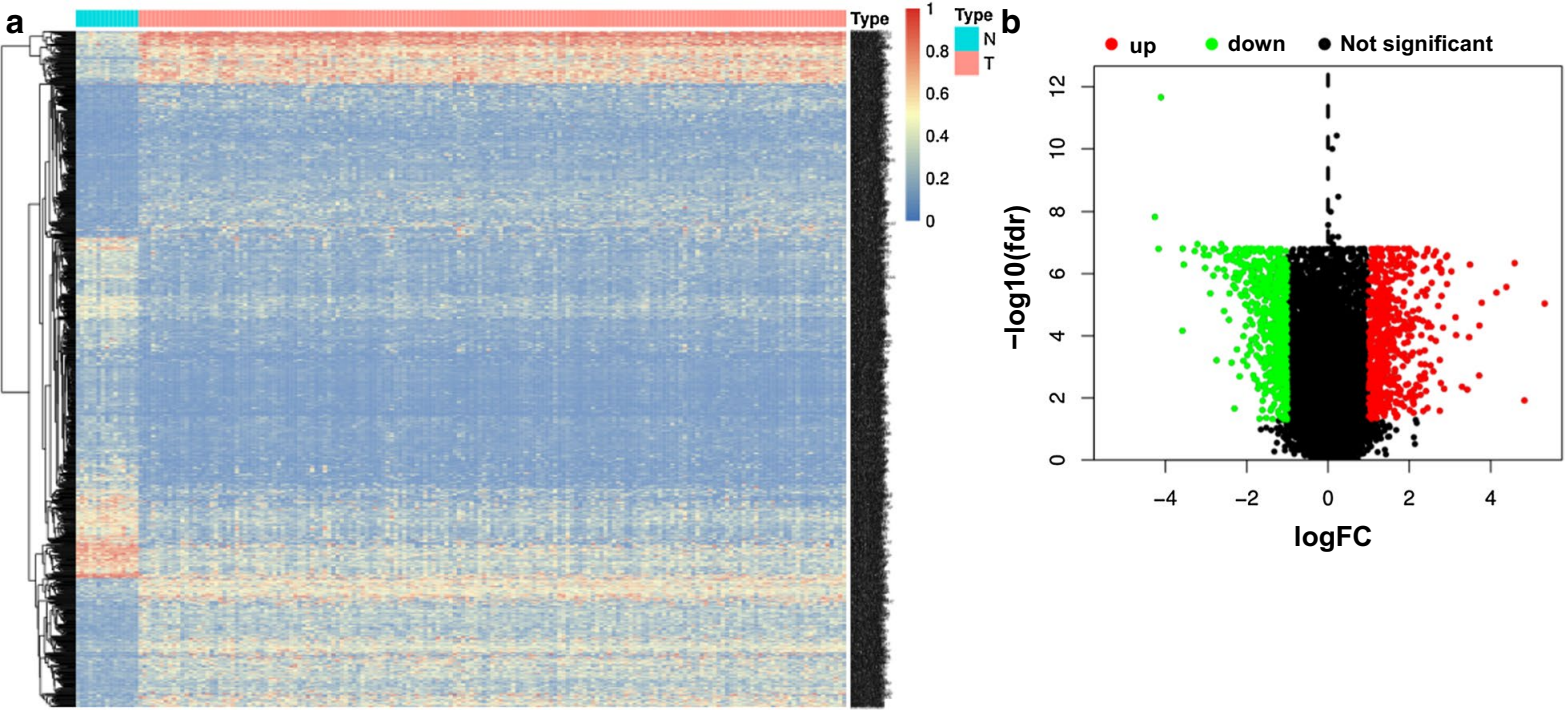
Identification of cancer-associated alternative splicing events (CAASs) in TNBC. **a** Heatmap of the CAASs between 169 cases of TNBC tissues and 15 cases of paracancerous tissues. **b** Volcano plot of CAASs identified in TNBC. The red and green points in the plot represent upregulated and downregulated CAASs, respectively

A flow chart summarizing the present work is shown in Fig. 1. We integrated the AS profiling of 169 TNBC tissues and 15 normal tissues to determine whether there were significant differences in AS events between tumour tissues and corresponding paracancerous normal tissues. Finally, we screened and identified 1194 differentially expressed CAAS events using the conditions of | log2fc |≥ 1 and FDR/adjusted *P* < 0.05. Meanwhile, a heat map (Fig. 2a) and a volcano plot (Fig. 2b) were generated to visualize CAAS events.

**Fig. 3:**
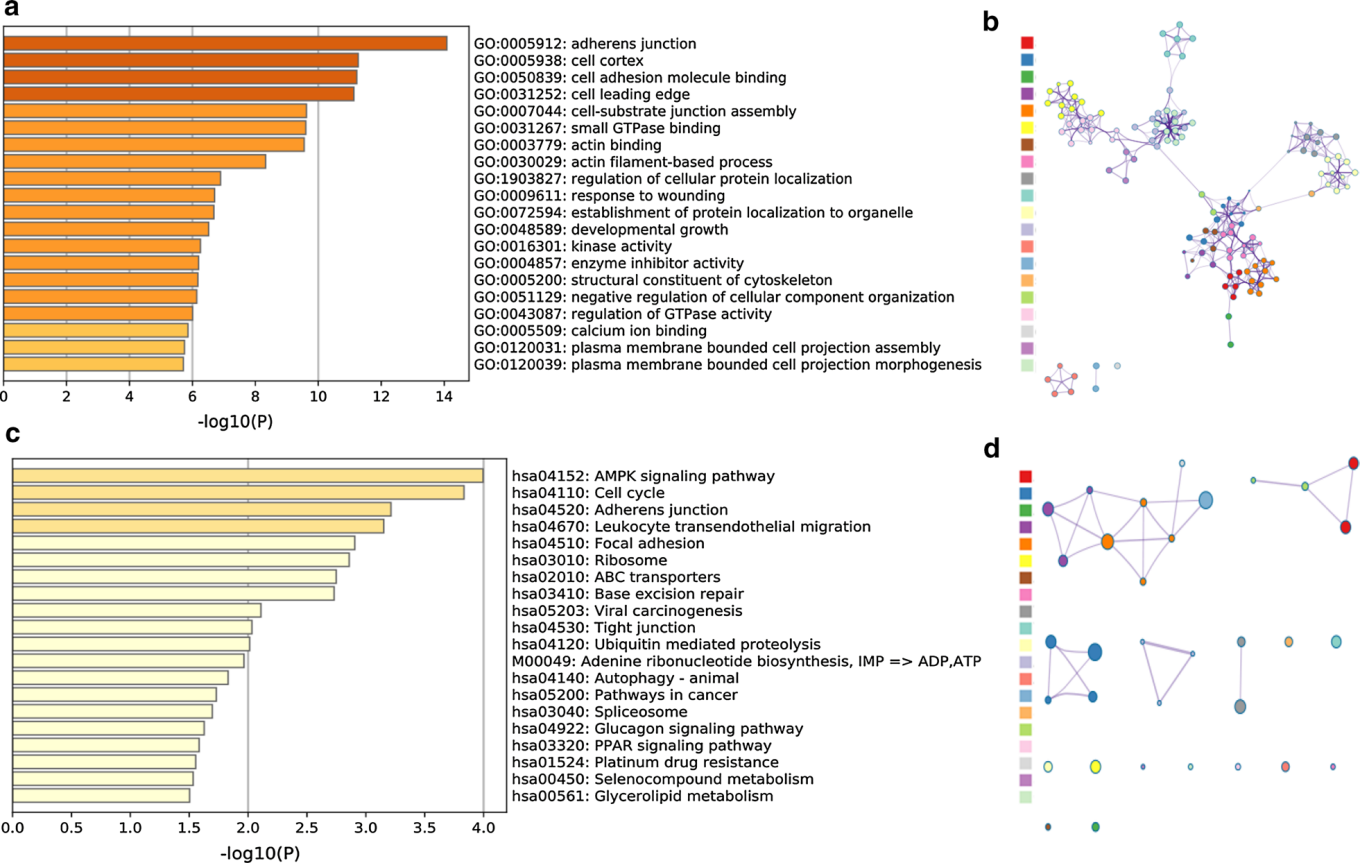
Functional enrichment analyses of the parent genes corresponding to nearly 1000 significant cancer-associated alternative splicing events (CAASs) in TNBC, including GO and KEGG. **a** Bar graph showing the top 20 results from the GO enrichment analysis. **b** GO enrichment analysis showing the gene networks. **c** Bar graph showing the top 20 results from the KEGG enrichment analysis. **d** KEGG enrichment analysis showing the enrichment of various pathways. The contents of a row of coloured squares on the left of Figure b and Figure d correspond in parallel to the contents of the right of Figure a and Figure c. *GO* Gene Ontology, *KEGG* Kyoto Encyclopedia of Genes and Genomes

Subsequently, enrichment analysis was performed, and the results are shown in Fig. 3. The top 20 results of enrichment pathways disclosed by biological processes in GO analysis included “adherens junction”, “cell cortex”, and “cell adhesion molecule binding”, among which the molecular functions of the cellular components (CC) accounted for the majority (Fig. 3a). The gene networks of the same top 20 results are displayed in Fig. 3b. Furthermore, it was suggested by KEGG enrichment analysis that some key pathways were relevant to the survival of TNBC patients, such as “MAPK signalling pathway”, “Cell cycle”, “Adherens junction”, “Leukocyte transendothelial migration” and “Focal adhesion” (Fig. 3c), whose interrelations of various pathways are illustrated in Fig. 3d. In addition, some KEGG pathways related to TNBC tumorigenesis and treatment difficulties were abundant, including “Viral carcinogenesis”, “Pathways in cancer”, “Autophagy” and “Platinum drug resistance”. Another interesting phenomenon was the enrichment of immune-related pathways, such as “Leukocyte transendothelial migration” and “PPAR signalling pathway”, which indicated that CAAS events in TNBC are also involved in immune microenvironment formation.

### Association between CAAS events and the tumour microenvironment

**Fig. 4:**
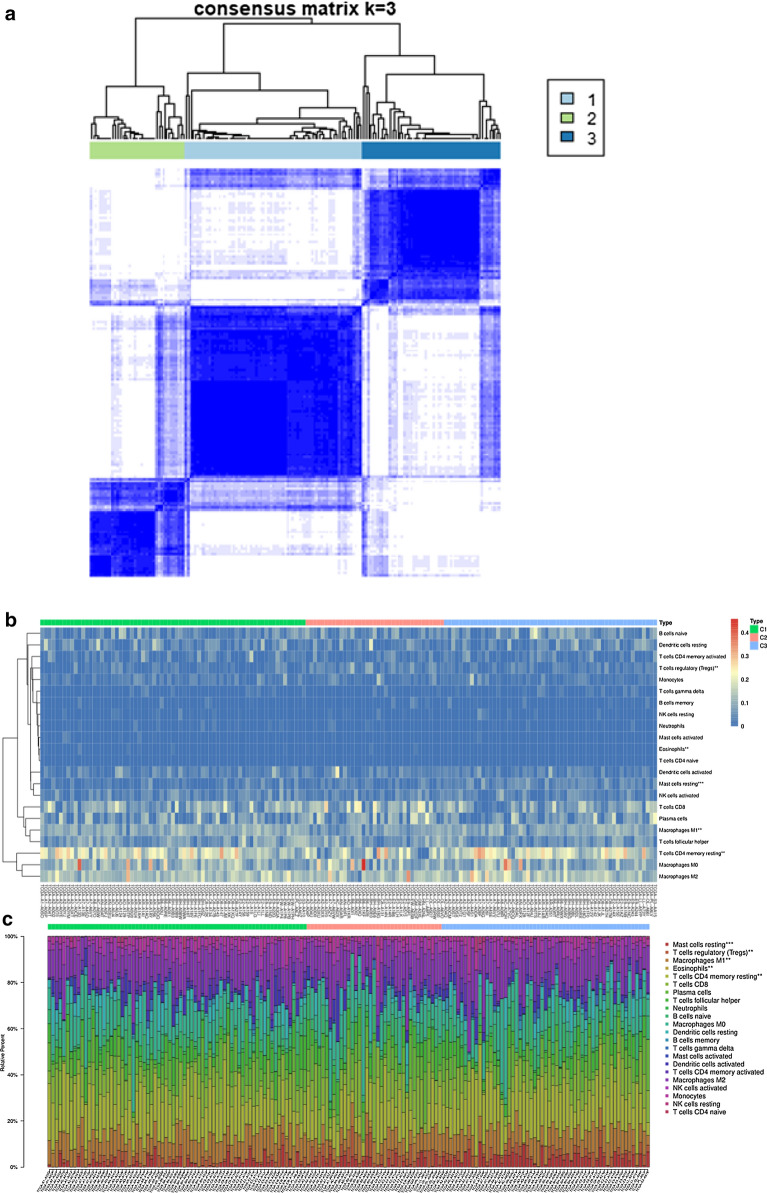

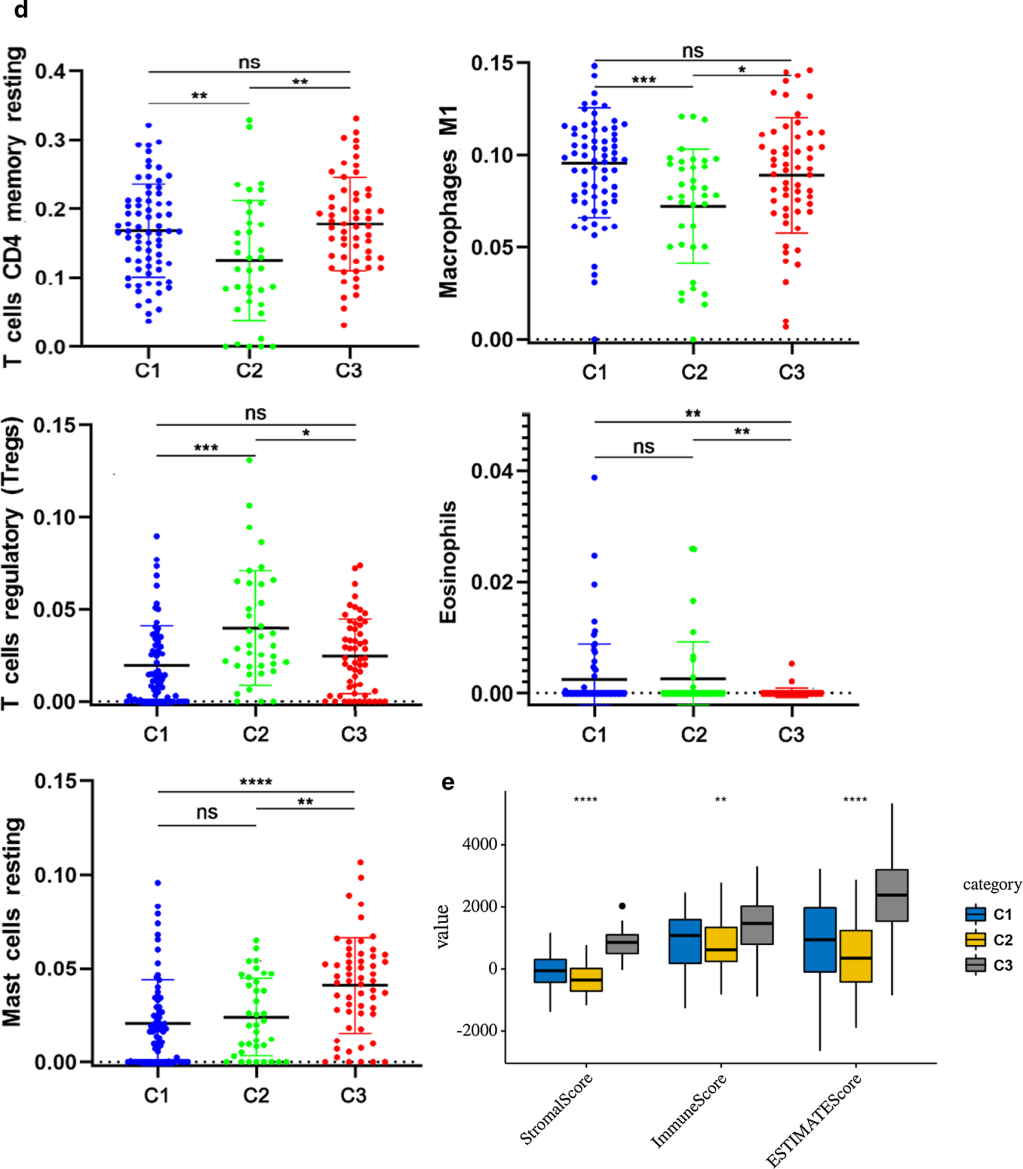
CAAS-based clusters significantly associated with immune microenvironment features (CIBERSORT *P* > 0.05). **a** Consensus clustering analysis identification of three clusters (samples, n = 165. The white (consensus value = 0, samples never clustered together) and blue (consensus value = 1, samples always clustered together) heatmap colours display sample consensus. **b** Heatmap of CAASs ordered by clusters showing differential expression of immune cells between patients. **c** Heatmap of CAASs ordered by clusters displaying the distribution of immune cells in each patient. The asterisk in the upper right corner of the immune cells indicates that the differential expression is significantly different. “*” represents *P* < 0.05; “**” represents *P < *0.01; “***” represents P < 0.001. **d** A demonstration of the specific expression of differentially expressed immune cells in clusters according to Fig. 4b and c. **e** Immune score and stromal score between CAAS-based clusters

These findings reminded us that the tumour-immune microenvironment turbulence in TNBC could be a prognostic factor for patients. Therefore, we further performed an unsupervised consensus analysis to assess the internal profile of the immune microenvironment based on CAAS events. We divided the patients into three clusters (Fig. 4a), among which there were significance differences in the expression levels of some immune cells, such as “Mast cell resting (*P *< 0.001)”, “T cell regulatory (*P* < 0.01)”, “Macrophages M1 (*P* < 0.01)”, “Eosinophils (*P* < 0.01)”, and “T cell CD4 memory resting (*P* < 0.01)” (Fig. 4b–d). The stimulation of immune cell infiltration in the C1 cluster was more than that in C2 cluster, which indicated that the generation of different immune states may have a great relationship with the classification of CASS clusters (Fig. 4d). Furthermore, the consensus matrix heatmap showed the difference of immune cell expression among different clusters (Fig. 4b, c). Besides, there was no difference in T cell CD4 memory resting, Macrophages M1 and T cell regulatory (adaptive immunity) between C1 and C3, but the difference was greatest in innate immune characteristics (Eosinophils and Mast cell resting) (Fig. 4d).

To further explore the underlying immunophenotypes among the three clusters, differences in the immune microenvironment among the CAAS-based clusters were further analyzed (Fig. 4E). The immune and stromal scores were calculated based on the ESTIMATE algorithm to quantify the presence of stromal cells and the infiltration of immune cells in tumor samples. We found that the immune phenotypes of the three clusters had a certain degree of heterogeneity, and we also noticed that C3 always had a higher immune and stromal score, followed by C1, and C2 was the lowest, which is similar to the situation of immune cell infiltration level (Fig. 4d).

### Regulatory network of CAAS events and SFs

**Fig. 5:**
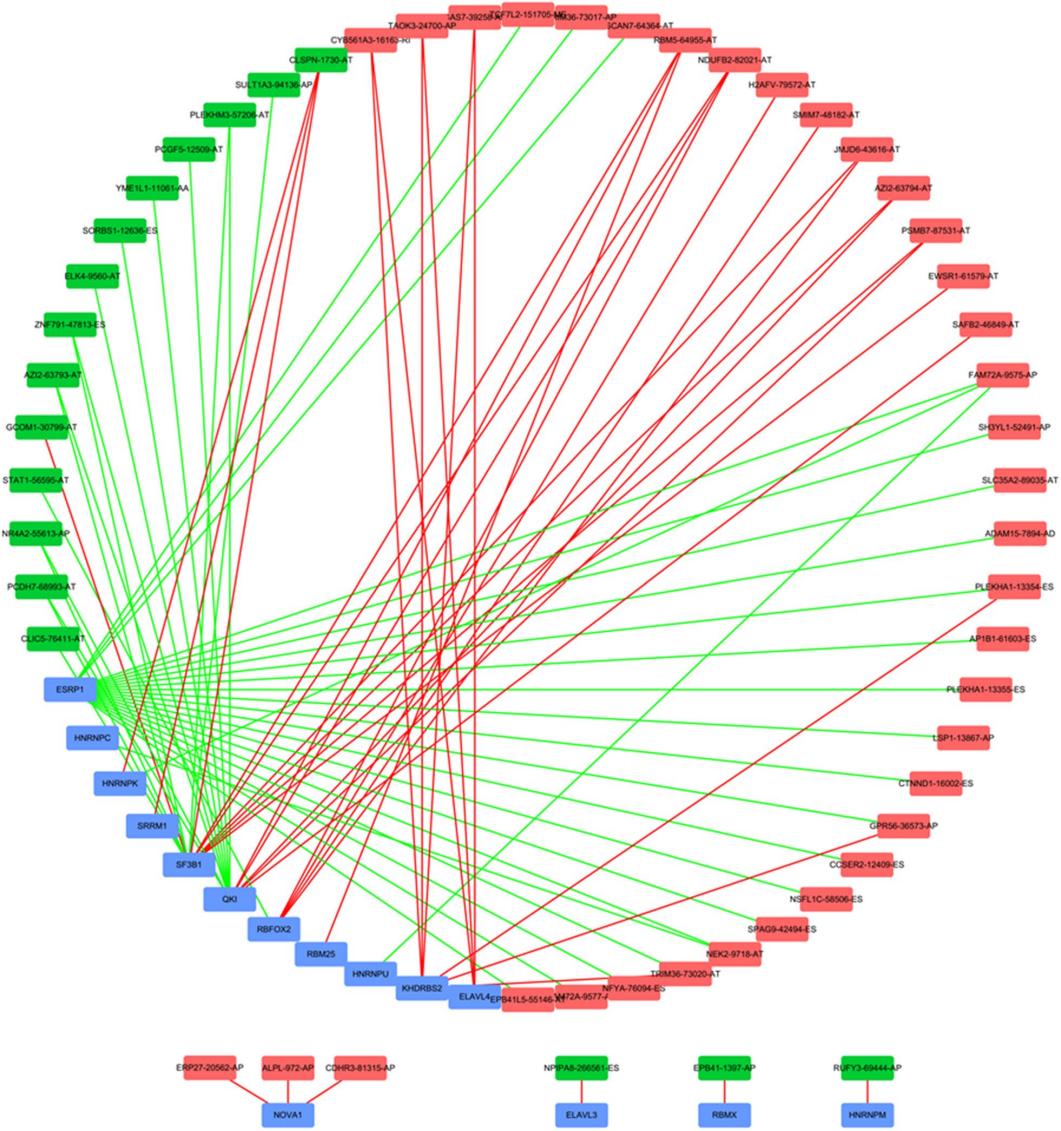
Splicing correlation network in TNBC. Expression of fifteen SFsb (bule dots) were positively (red lines)/negatively (green lines) correlated with PSI values of CAAS events with favorable prognosis (green dots) or CAAS events with inferior prognosis (red dots)

It is well known that SF plays an important regulatory role in the change and formation of AS events. For the purpose of exploring their potential connections, we downloaded a total of 71 SFs data from the SpliceAid2 database. Then Pearson correlation analysis was performed to determine the correlation between the PSI value of CAAS events and SF expression. The significant correlations (|r| ≥ 0.5, *p* < 0.001) were selected to construct the regulation network (Fig. 5). The regulation network consists of 53 CAAS events, of which 36 were adverse AS events (red dots) and 17 were favorable AS events (green dots), were significantly correlated with the 15 SFs (blue dots). We can find that most of the SFs were correlated with multiple AS events and played opposite roles in regulating different AS events. Similarly, a part of the AS events could be regulated by different SFs. This phenomenon partly explained that the same transcript can produce multiple different splicing events. Interestingly, the majority of the adverse AS events were positively correlated with SF expression (red lines), whereas the majority of favorable AS events were negatively correlated with SF expression (green lines).

### Identification of survival-related CAAS events in TNBC

**Fig. 6:**
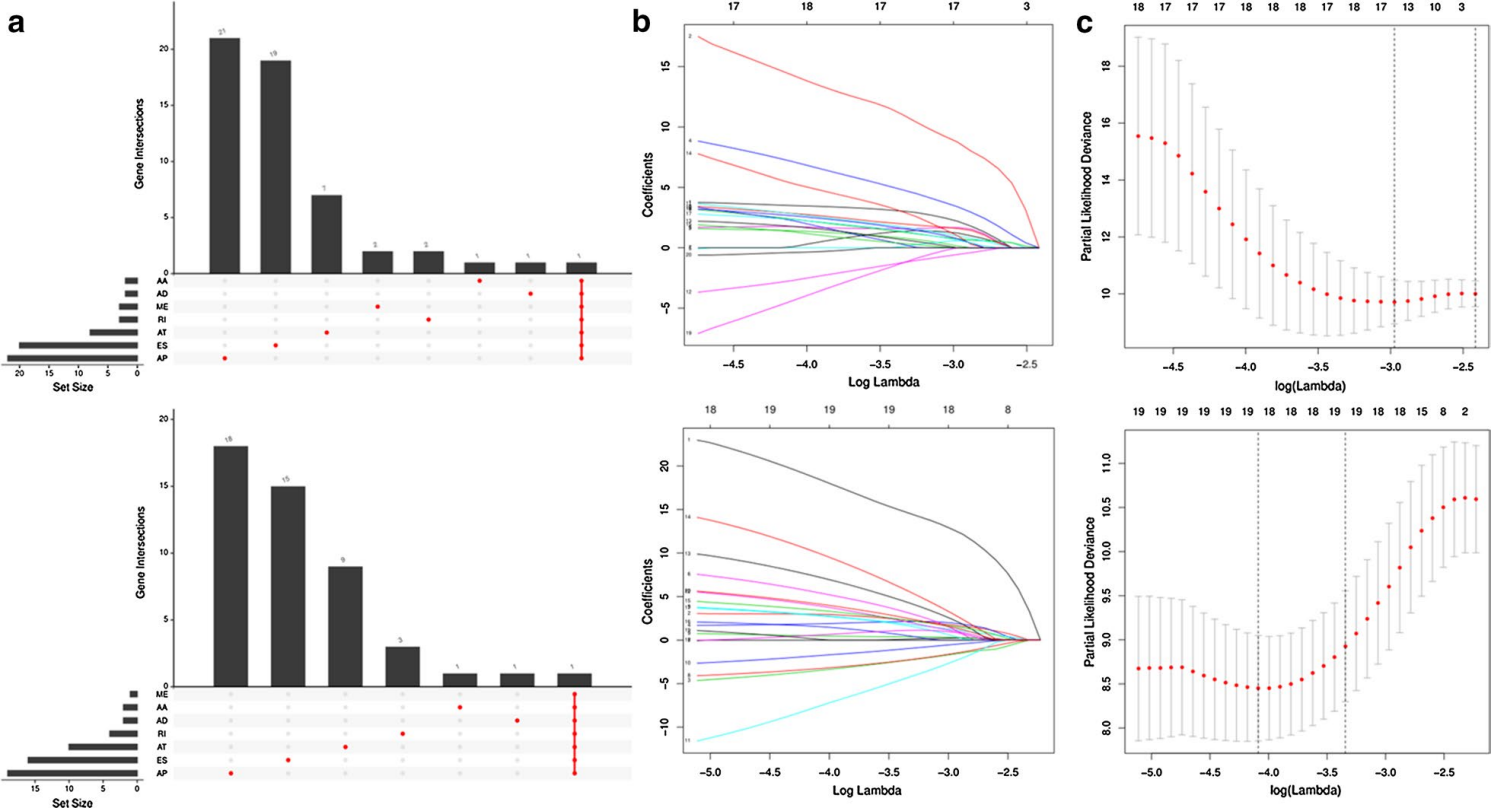
Selection of the optimal survival-related AS events in TNBC used for construction of the final prediction model by using Upset plot and LASSO Cox regression based on OS and PFS. **a** Upset plots of the intersections between the seven types of survival-related AS events. **b** LASSO coefficient profiles of the candidate survival-related AS events. A coefficient profile plot was produced against the logλ sequence. LASSO coefficient profiles of the candidate survival-related AS events. **c** Dotted vertical lines were drawn at the optimal values by using the minimum criteria. The upper parts and the bottom are related to OS and PFS, respectively. *AS* alternative splicing, *TNBC* triple negative breast cancer, *LASSO* least absolute shrinkage and selection operator, *OS* overall survival, *PFS* progression-free survival

By performing univariate Cox regression analysis, a total of 48 CAAS events were identified as OS-associated CAAS events, while 48 CAAS events were identified as PFS-associated CAAS events. Considering that a gene may have two or more AS events that are significantly related to the prognosis of TNBC, the UpSet plot maybe be the best choice to show the distribution of survival-related AS events in the seven AS types and visualize the intersection set from two study endpoints of OS and PFS (Fig. 6a; the upper and lower parts are related to OS and PFS, respectively). Both figures agreed that AP (alternate promoter) is the most common event related to the prognosis of TNBC.

### Establishment and assessment of the prognostic signature for TNBC patients

**Fig. 7:**
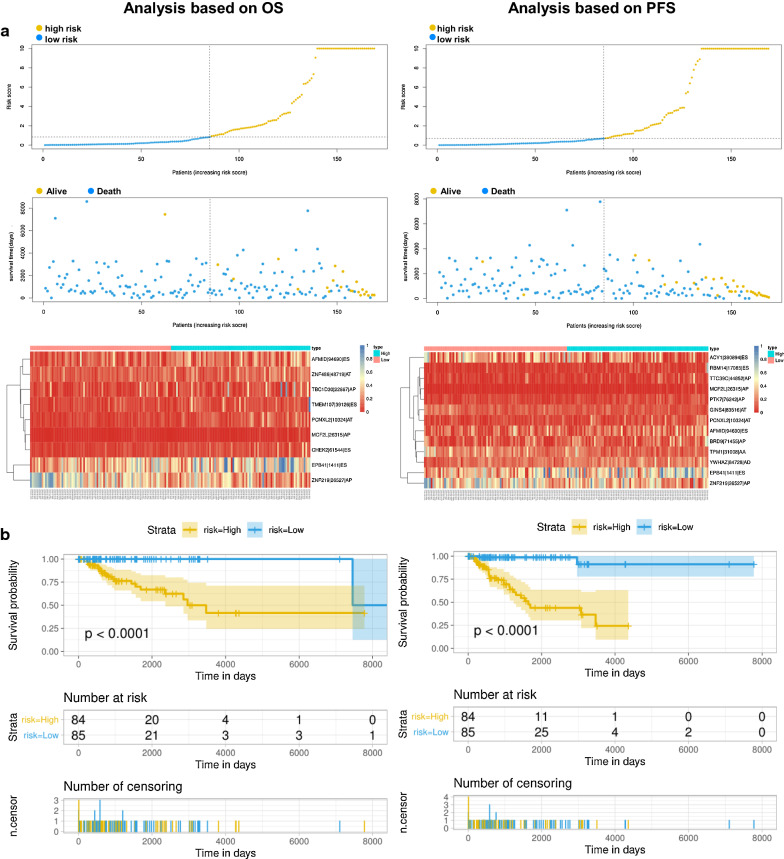

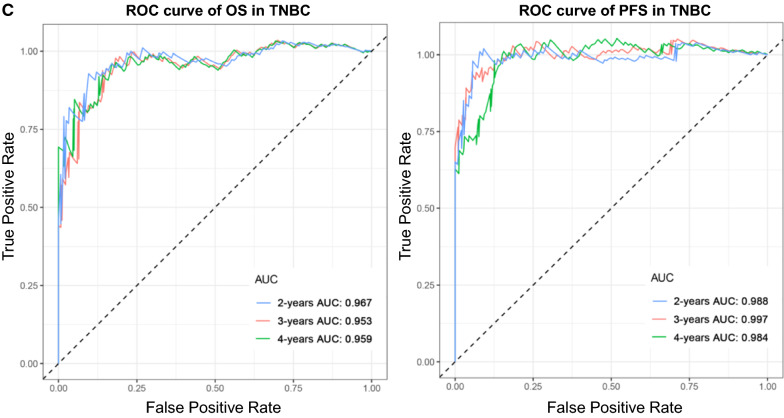
Analysis of the AS multivariate prognostic model in TNBC from two aspects of OS and PFS. **a** Upper part shows the risk score curves for survival-AS events; the middle shows survival status and survival times of TNBC patients ranked by risk score. The black dotted line represents the optimum cut-off point dividing patients into low- and high-risk groups; the bottom shows the heat map of the PSI value of survival-AS events. Colours from red to blue indicate decreasing PSIs from high to low. **b** Upper part shows the Kaplan–Meier curves for the high- and low-risk groups; the middle shows the number of living patients variation with time in the high- and low-risk groups; the bottom shows the number of censoring variation with time in the high- and low-risk groups. Blue colour represents low-risk group data, whereas yellow colour represents high-risk group data. **c** The ROC curves of prognostic models at 2, 3 and 4 years. Blue colour represents 2 years, red colour represents 3 years, and green colour represents 4 years. The columns on the left represent OS, whereas the columns on the right represent PFS. *PSI* percent spliced in, *ROC* receiver operator characteristic, *AUC* area under the curve

After conducting univariate regression analysis, LASSO regression was performed to select the optimal survival-related AS events to construct the prediction models to avoid model overfitting based on OS (Additional file 1) and PFS (Additional file 2), respectively (Fig. 6b, c). Meanwhile, the risk scores of each TNBC patient were calculated, and all patients were divided into low- and high-risk groups bounded by the median risk score (Fig. 7a; the columns on the left represent OS, whereas the columns on the right represent PFS). K-M curves and log-rank tests were plotted to explore the relationship between risk score and survival status. The survival probability of low-risk patients was higher than that of high-risk patients; in other words, high-risk patients had a higher mortality rate, exactly as illustrated in Fig. 7b (*P* < 0.0001). We then applied ROC analysis to compare the predictive power of these prognostic models, which showed a robust and significantly improved performance, whose AUCs of ROC in 2, 3, and 4 years were all greater than 0.900 (Fig. 7c). Interestingly, we also found four overlapping AS events (AFMIDl94690lES, MCF2Ll26315lAP, EPB41l1411lES, ZNF219l26517lAP), with significant differences found in the analysis of two different study endpoints, OS and PFS simultaneously, indicating that they are most likely independent prognostic factors (Fig. 7a).

### AS-clinic nomogram for predicting individual prognosis of TNBC patients

**Fig. 8:**
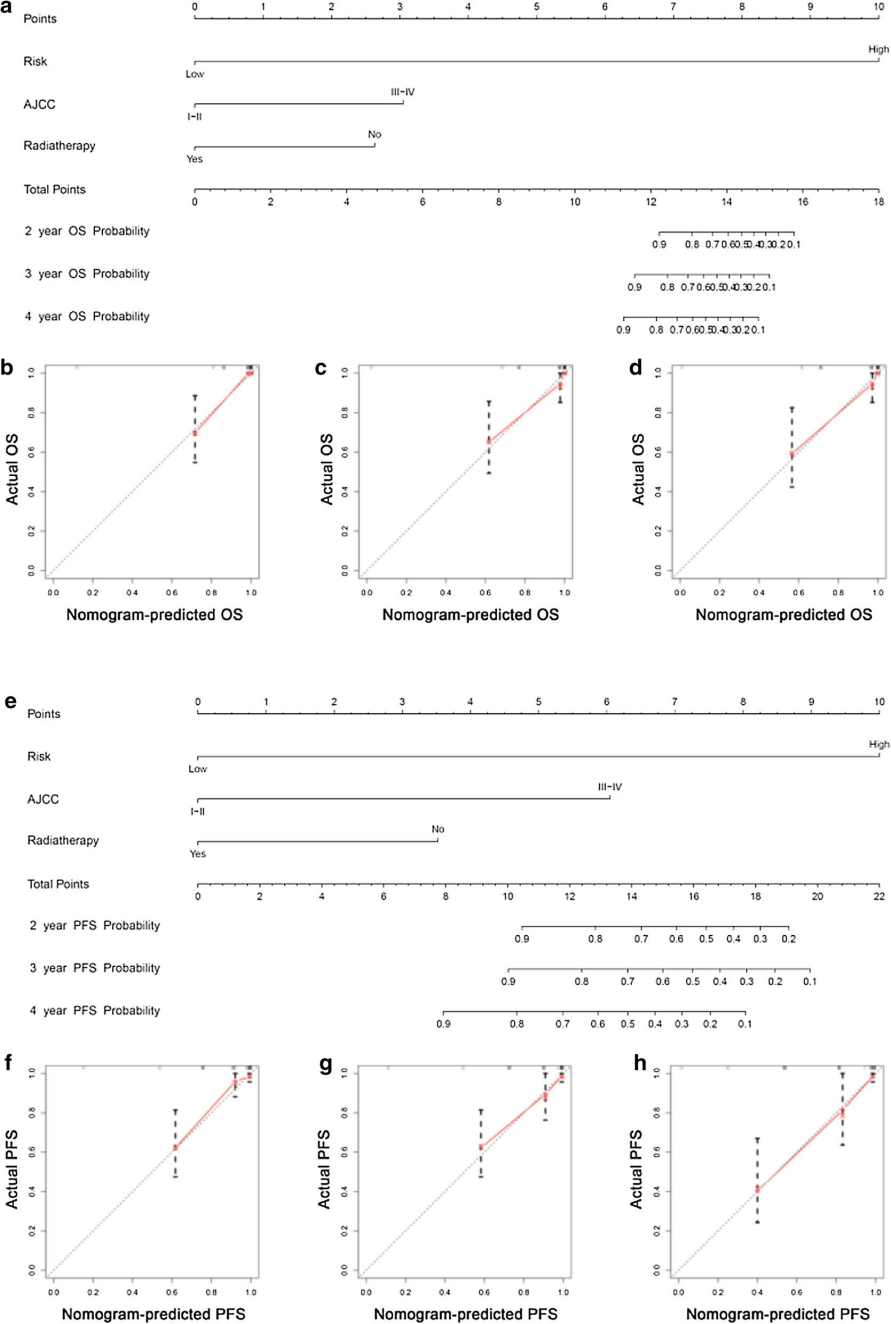
AS-clinic nomograms for predicting the individualized survival rates of TNBC patients for two aspects of OS and PFS. **a**, **e** Construction of AS-clinic nomograms for TNBC patients to predict 2, 3, and 4 year OS (**a**) and PFS (**e**), which were incorporated with 3 variables, including risk, AJCC and radiotherapy. **b**–**d**, **f**–**h** Calibration plots of the AS-clinic nomograms in terms of agreement between nomogram-predicted and observed 2, 3, and 4 year outcomes of the TNBC cohort, including OS (**b**–**d**) and PFS (**f**–**h**). The 45º dashed line represents the ideal performance. The actual performances of the model are represented by the red lines, and the figures from left to right show the 2, 3, and 4 year results

The results of univariate Cox analysis of clinic characteristics, including OS and PFS, are displayed in Table 1, which showed that risk, AJCC, radiotherapy, and N stage were OS-related factors and that risk, AJCC, T stage, N stage, M stage, and radiotherapy were PFS-related variables. Then, with the forward stepwise selection on optimizing AIC applied based on multivariate Cox analysis (Table 2), we finally chose three variables, including risk, AJCC and radiotherapy, for developing OS and PFS nomograms (Fig. 8a, Fig. 8e). There was good agreement between the predicted value and the actual value, which was confirmed by the calibration curve of these nomograms for the probability of survival at 2, 3, or 4 years (Fig. 8b–d, Fig. 8f–h), respectively. The C-index for the OS nomogram was 0.939 (95% CI, 0.900–0.978), whereas the C-index for the PFS nomogram was 0.867 (95% CI, 0.777–0.957). These outcomes revealed that the nomogram had major clinical application value in predicting long-term survival probability.

**Table 1:** Screening clinical variables related to prognosis by univariate analysis in TNBC cohort

	OS				PFS			
	HR	95% CI		*P*	HR	95% CI		*P*
Risk	24.311	3.264	181.084	0.002	20.068	4.771	84.404	0.000
Age	1.018	0.985	1.052	0.288	1.006	0.978	1.034	0.699
AJCC								
I-II				0.000				0.000
III-IV	6.079	2.492	14.829	0.000	4.879	2.259	10.535	0.000
NA	1.203	0.138	10.462	0.867	1.300	0.171	9.866	0.800
M								
0				0.323				0.001
1	3.478	0.677	17.864	0.135	18.603	3.882	89.139	0.000
NA	1.223	0.280	5.339	0.789	1.219	0.364	4.084	0.748
N	4.468	1.746	11.430	0.002	2.994	1.423	6.296	0.004
T								
1-2				0.190				0.131
3-4	2.574	0.931	7.116	0.068	2.425	1.026	5.735	0.044
NA	0.000	0.000	0.000	0.981	0.000	0.000	0.000	0.983
Radiotherapy								
No				0.000				0.001
Yes	0.315	0.121	0.826	0.019	0.439	0.173	1.111	0.082
NA	0.060	0.017	0.207	0.000	0.159	0.060	0.419	0.000
Race								
Asian				0.304				0.791
White	0.192	0.023	1.571	0.124	0.363	0.046	2.834	0.334
Black	0.361	0.044	2.973	0.344	0.435	0.054	3.521	0.435
NA	0.000	0.000	0.000	0.985	0.000	0.000	0.000	0.980

*HR* hazard ratio, *AJCC* American Joint Committee on Cancer, *NA* not applicable

**Table 2:** Detailed information of specific AS events involved in final prognostic model by multivariate analysis

	OS				PFS			
	HR	95% CI		*P*	HR	95% CI		*P*
Risk	32.503	2.105	501.818	0.013	0.040	0.005	0.301	0.002
AJCC								
I-II				0.000				0.000
III-IV	16.561	4.728	58.007	0.000	6.939	2.786	17.282	0.000
NA	0.273	0.026	2.876	0.280	11.715	0.674	203.462	0.091
Radiotherapy								
No				0.000				0.002
Yes	0.318	0.102	0.995	0.049	0.543	0.198	1.490	0.236
NA	0.023	0.005	0.113	0.000	0.164	0.053	0.504	0.002

*HR* hazard ratio, *AJCC* American Joint Committee on Cancer, *NA* not applicable

## Discussion

Recently, great breakthroughs have been made in the study of the potential significance of AS profiling in tumour biology with the enormous progress of high-throughput sequencing technology. Although the prognostic capacity of AS events has been widely confirmed in many cancers [21–24], the comprehensive profiling of AS events in TNBC patients is still lacking. In this study, we obtained and integrated clinical and AS events data from the cancer genome atlas (TCGA) and the SpliceSeq database to obtain 1194 CAAS events and identified the AS events related to survival. Moreover, two CAAS event-based signatures were generated. It was found that the prognosis of patients in the low-risk group was better than that in the high-risk group. Additionally, CAAS-based risk, AJCC and radiotherapy were identified as independent OS- and PFS-related variables, which were incorporated into nomograms, and the results indicated that the two nomograms can serve as effective tools for clinical practice with TNBC patients.

In the present study, two CAAS event-based signatures were developed and showed a favourable predictive capacity. Interestingly, five overlapping AS events were identified among the splicing events, with significant differences based on OS and PFS simultaneously, including AFMIDl94690lES, MCF2Ll26315lAP, EPB41l1411lES, ZNF219l26517lAP and PCNXL2|10324|AT, suggesting that they are the most likely independent prognostic factors. AFMID (arylformamidase) is located on chromosome 17q25.3 and encodes arylformamidase, a control enzyme in tryptophan metabolism. Consistent with our findings, Krainer et al. also found that specific splicing events of the AFMID gene are significantly associated with survival in hepatocellular carcinoma (HCC) patients. The conversion of the AFMID subtype represents a new regulatory step in tryptophan/kynurenine metabolism, revealing the disruption of neonatal NAD + biosynthesis in hepatocytes in the early stages of tumour development [32]. In addition, NAD + is an important coenzyme in the energy metabolism of eukaryotic cells [33]. Previous studies have shown that NAD + supplementation can extend the life span of mice [34]. However, the ratio of NAD + /NADH in cancer cells is very low, and they maintain enough NAD + by converting pyruvate to lactate to achieve high-speed glycolysis while shutting down other sources of NAD + production [35]. In summary, repairing AFMID splices may lead to the augmentation of NAD + production and DNA repair. Therefore, it is expected that AFMID splicing could become a therapeutic target and a source of new cancer drugs after additional research.

Regarding PCNXL2 (pecanex-like 2), namely FLJ11383|KIAA0435, a previous report suggested that it might have a certain influence on the tumorigenesis of colorectal carcinomas with high microsatellite instability [36], which may be a new breakthrough point for immunotherapy with the progress of genomics research and the maturation of protein antibody preparation technology. Nevertheless, the main focuses of recent studies were established based on the correlation of PCNXL2 as a novel susceptibility locus of thyroid cancer [37, 38], which was speculated to be related to the prognosis of thyroid cancer. In addition, there is a broad consensus that the emergence of chemotherapy resistance is also a major problem affecting the therapeutic effect. MC2L is one of the guanine nucleotide exchange factors, which may link the potential signalling pathway through RAC1, RHOA and CDC42, also named DBS/DBLs Big Sister, belonging to the DBL family [39]. Research has shown that MCF2L/DBS MCF2L may play an important role in gemcitabine resistance of primary pancreatic cancer patients [40], which may provide some explanations for the causes of TNBC chemotherapy resistance. It should be noted that the EPB41 gene encodes Erythrocyte Membrane Protein Band 4.1, which belongs to the family of cytoskeletal proteins that play important roles in maintaining normal cell morphology and cell adhesion, migration, division, and intercellular signalling [41–43]. Many studies have revealed the capacity of EPB41 to predict the prognosis of various cancers and its critical role in the development of tumours, such as breast cancer [44], meningiomas [45], prostate cancer [46], and hepatocellular carcinoma [47]. Together, this evidence supports the biological relevance of EPB41 in tumour biology. Our findings are mostly consistent with the above results, but further research is still needed.

Moreover, we also found that the parent genes of CAAS events were significantly enriched in several functions and pathways, revealing the potential molecular functions and signalling pathways associated with TNBC progression and treatment difficulties. In addition, some immune-related mechanisms were also identified, such as “Leukocyte transendothelial migration” and “PPAR signalling pathway”. Previous research had proven that PPARγ (peroxisome proliferator-activated receptors) is a key regulator of lipid and glucose metabolism in many cell types, with robust anti-inflammatory activity in immune cells [48, 49]. Moreover, PPARγ^High^ /RXRα^S427F/Y^ interferes with CD8 + T cell infiltration and participates in immunotherapy resistance. Knockdown of PPARγ or RXRα and inhibition of PPAR can restore immune surveillance and sensitivity to immunotherapy [50]. Inspired by these findings, we further investigated potential interactions between CAAS events and the tumour microenvironment. Unsupervised clustering analysis was adopted and found that the expression levels of some immune cells between clusters, such as “Mast cell resting (*P* < 0.001)”, “T cell regulatory (*P* < 0.01)”, “Macrophages M1 (*P < *0.01)”, “Eosinophils (*P* < 0.01)”, and “T cell CD4 memory resting (*P* < 0.01)”, were significantly different, demonstrating that differences in CAAS could lead to changes in the tumour immune microenvironment. Previous studies have shown that high infiltration of M1 [51] and CD4 T cells, E0 expression [52], mast cell expression [53], and low Treg expression [54] or other states are the best state of hot tumours, which can increase the efficacy of immunotherapy. What’s more, the main difference between the three clusters was the infiltration level of innate and acquired immune cells, which was verified by differential analysis of the underlying immunophenotype and immune microenvironment.In addition, we also discovered the heterogeneity of the immune microenvironment in TNBC based on the consensus matrix heatmap, which could to some extent explain the clinical phenomenon that PD-1 / PD-L1 immunotherapy had different effects on TNBC patients.

Additionally, the potential involvement of splicing factors was also considered in our analysis. Splicing correlation network revealed that the relationship between SFs and AS was not a one-to-one correspondence, but had multiple interactions, which partly explained the diversity of AS events. And the other thing that was interesting was that the majority of the adverse AS events were positively correlated with SF expression, whereas the majority of favorable AS events were negatively correlated with SF expression, which may provide a way to elucidate the potential mechanism of splicing pathways involved in patient survival. However, due to the limited number of acquired SFs, we were unable to screen out survival-related data for study. Therefore, the survival regulation mechanism of the SF‐AS network was not yet clear and in-depth functional investigation was necessary.

Our study was the first to analyse the AS profile in TNBC from multiple perspectives, selected the most relevant prognostic factors, established a prognostic model with high accuracy, and further accurately predicted the individual survival rate of TNBC patients. Moreover, the immune microenvironment turbulence in the TNBC population was revealed from the perspective of immunology. However, it is inevitable that our research still has several limitations that we should consider. First, the TNBC-related data we obtained through the TCGA database included only 169 cases, the sample size was not large enough, and our results were only verified by the TCGA dataset without additional external datasets. Second, adjacent normal tissues do not necessarily represent the origin of tumour cells, whose changes in expression may not be a requirement for splicing variants to function [55]. In addition, our study is based on pure bioinformatics analysis and lacks clinical validation. Therefore, further studies of the biological role and molecular mechanism of AS events in TNBC tumorigenesis are needed.

In summary, this study has enriched AS profiling research on breast cancer, simultaneously and for the first time unveiling the characteristics of AS events in TNBC. In addition, we established a powerful prognostic model to accurately predict the prognosis of TNBC patients. More importantly, this comprehensive analysis based on differentially expressed AS events has enhanced our understanding of AS events promoting tumorigenesis and development, which are more likely to be potential clinical biomarkers and therapeutic targets and provide guiding significance for future basic research and clinical work.

## Conclusion

Based on the comprehensive bioinformatics analysis, our study was the first to showed that AS events were closely linked to tumorigenesis and the immune microenvironment in TNBC. Further, we established the prediction model with good performance in basis of survival-related AS events. Besides, we identified five independent prognostic factors, including AFMIDl94690lES, MCF2Ll26315lAP, EPB41l1411lES, ZNF219l26517lAP and PCNXL2|10324|AT, each worthy of further investigation.These findings provided insight into the connection between AS events and TNBC.

## Supplementary information

**Additional file 1:** The optimal OS-related AS events was selected by LASSO regression for constructing the prediction models.

**Additional file 2:** The optimal PFS-related AS events was selected by LASSO regression for constructing the prediction models.

## Data Availability

The datasets generated during and/or analysed during the current study are available from the corresponding author on reasonable request.
